# Single-Cell RNA Sequencing of Visceral Adipose Tissue Leukocytes Reveals that Caloric Restriction Following Obesity Promotes the Accumulation of a Distinct Macrophage Population with Features of Phagocytic Cells

**DOI:** 10.20900/immunometab20190008

**Published:** 2019-07-19

**Authors:** Ada Weinstock, Emily J. Brown, Michela L. Garabedian, Stephanie Pena, Monika Sharma, Juan Lafaille, Kathryn J. Moore, Edward A. Fisher

**Affiliations:** 1 Department of Medicine, Division of Cardiology, Marc and Ruti Bell Program in Vascular Biology, NYU School of Medicine, New York, NY 10016, USA; 2 Department of Microbiology and Immunology, NYU School of Medicine, New York, NY 10016, USA

**Keywords:** adipose tissue, obesity, caloric-restriction, weight loss, leukocytes, macrophages, heterogeneity, single-cell RNA-seq, phagocytosis

## Abstract

Obesity can lead to type 2 diabetes and is an epidemic. A major contributor to its adverse effects is inflammation of the visceral adipose tissue (VAT). Life-long caloric restriction (CR), in contrast, results in extended lifespan, enhanced glucose tolerance/insulin sensitivity, and other favorable phenotypes. The effects of CR following obesity are incompletely established, but studies show multiple benefits. Many leukocyte types, macrophages predominantly, reside in VAT in homeostatic and pathological states. CR following obesity transiently increases VAT macrophage content prior to resolution of inflammation and obesity, suggesting that macrophage content and phenotype play critical roles. Here, we examined the heterogeneity of VAT leukocytes and the effects of obesity and CR. In general, our single-cell RNA-sequencing data demonstrate that macrophages are the most abundant and diverse subpopulation of leukocytes in VAT. Obesity induced significant transcriptional changes in all 15 leukocyte subpopulations, with many genes showing coordinated changes in expression across the leukocyte subpopulations. Additionally, obese VAT displayed expansion of one major macrophage subpopulation, which, in silico, was enriched in lipid binding and metabolic processes. This subpopulation returned from dominance in obesity to lean proportions after only 2 weeks of CR, although the pattern of gene expression overall remained similar. Surprisingly, CR VAT is dominated by a different macrophage subpopulation, which is absent in lean conditions. This subpopulation is enriched in genes related to phagocytosis and we postulate that its function includes clearance of dead cells, as well as excess lipids, contributing to limiting VAT inflammation and restoration of the homeostatic state.

## INTRODUCTION

Adipose tissue (AT) contains diverse leukocyte populations, of which the most predominant are macrophages (Møs), which constitute ~5% of cells in the AT of lean mice and humans [1]. Other leukocytes previously described in the AT include dendritic cells (DCs), T cells, natural killer (NK) cells, innate lymphoid cells (ILCs), eosinophils, *etc.* (reviewed in [1]). Previous work has demonstrated that obesity results in quantitative and qualitative changes in the leukocyte compartment. For instance, in the obese AT, Møs increase in abundance to account for ~50% [2] of cells and T cell abundance also increases ~3 fold [3].

Although it is well-established that there are quantitative changes in the leukocyte composition in obesity, there is considerable ambiguity in the field regarding the qualitative changes of the different populations. Some studies suggest that in obesity, several of the visceral AT (VAT) leukocyte populations, such as Møs [4,5], T cells [6,7] and DCs [8,9] exacerbate the inflammatory response and cause insulin resistance. Other work suggests that Møs and DCs are anti-inflammatory in the lean VAT and undergo a phenotypic switch to become pro-inflammatory in obesity, via recruitment of CCR2^+^ monocytes to the VAT and differentiation into inflammatory Møs [10] and DCs [9]. Still, other investigations suggest that the metabolic state of the VAT itself regulates leukocyte abundance and function. For example, the breakdown of lipids (via lipolysis) and secretion of fatty acids by adipocytes during fasting, lipodystrophy and pharmacological activation of adrenergic receptors were shown to rapidly increase leukocyte content in the VAT [11–13].

In general, obese VAT has more leukocytes than lean VAT. Somewhat counterintuitively, weight loss following obesity has also been shown to, at least transiently, elevate AT leukocyte counts in both mice [13] and humans [14], due to local proliferation [15] and increased migration in response to adipocyte lipolysis [13]. However, it is not yet clear what changes occur in leukocyte subtypes in the VAT following weight loss. Caloric restriction (CR) of obese mice was shown to induce rapid AT macrophage (ATM) accumulation, peaking at 3 days post treatment and gradually decreasing thereafter, up to day 42 [13]. In another mouse model of weight loss, it has been shown that feeding mice chow diet following diet-induced obesity results in a sustained inflammatory signature of ATMs [15]. Similarly, weight loss following bariatric surgery modulates the abundance of different leukocyte populations in the subcutaneous adipose tissue, while maintaining the expression levels of several pro-inflammatory cytokines, as measured in whole tissue extracts [16].

Most previous investigations of VAT leukocytes have involved selection of cells according to expression of surface markers, resulting in a biased sampling of known cell types [4,17–19]. These strategies have primarily allowed for the characterization of 2 major subtypes of ATMs, which can be delineated via their surface expression of CD11c. More recently, 8 mononuclear phagocyte populations were described using cell sorting and bulk RNA sequencing (RNA-seq), showing that obesity does not promote a clear inflammatory signature [20]. With single-cell RNA-seq (scRNA-seq), it is now possible to explore the heterogeneity of cellular populations in an unbiased manner [21]. scRNA-seq of 37 individual Mø cells isolated from obese VAT was reported recently, showing 2 main Mø subtypes that can be delineated via their CD9 expression [17]. However, this study used pre-selected markers (CD11b^+^, CD64^+^, F4/80^+^ and Ly6c^−^) to purify Møs, and had very few cells (37 Møs), hindering the ability to identify diverse or more rare populations with any degree of certainty.

We hypothesized that the heterogeneity of VAT leukocytes in general, and Møs in particular, is greater than appreciated previously. To test this hypothesis, we employed scRNA-seq to describe mouse VAT leukocyte heterogeneity in obesity and following a brief period of weight loss. Our analysis found 15 distinct leukocyte subpopulations, of which 7 are Møs. Obesity induced marked alterations in both gene expression and the proportion of VAT leukocytes subpopulations, compared with leaness. Following a brief period of CR, cells largely maintained the gene expression profile of the obese state, and promoted reversion of the cellular abundance of some subpopulations to lean proportions (among them ILC2/Treg and Major Møs). However, most strikingly, CR induced the accumulation of a Mø subpopulation enriched in genes associated with phagocytosis and endocytosis (thus termed Phagocytic Møs). We hypothesize that these Phagocytic Møs are responsible for clearance of apoptotic/necrotic cells in the VAT, and we will present evidence from an independent and direct examination of VAT in CR mice consistent with this.

## MATERIALS AND METHODS

### Animal Studies

All animal procedures were approved by the NYU School of Medicine IACUC Committee (160725–01, approved 7/21/2016). Six-week old C57BL/6J male mice were purchased from Jackson Laboratories and acclimated for 2 weeks in an SPF facility. Mice were maintained in a temperature-controlled (25 °C) facility with a 12-h light/dark cycle. Mice were placed on a diet containing 60% kcal from fat and 0.3% from cholesterol (Research Diets) and injected weekly with 5 mg/kg low-density lipoprotein receptor antisense oligonucleotide for 24 weeks, as described previously [22]. The antisense oligonucleotide was generously provided by Ionis Pharmaceuticals. Mice were given free access to water and food and on weeks 21–23 daily food consumption was measured by weighing the food every other day. On week 24 the obese group was harvested and the calorically restricted mice were given daily 70% of their *ad libitum* consumption of the same high-fat diet.

Burl *et al.* generated single cell suspension from 8 week old male mice and used all stromal vascular cells for single cell RNA-seq (without prior cell sorting) using the 10× Genomics platform [23]. For the purposes of this study, 2268 cells with detectable expression of CD45 were selected for further analysis.

### Adipose Tissue Isolation and Digestion

Mice were euthanized and perfused with 10 mL saline solution to remove peripheral blood and ensure that leukocyte populations found are those residing in the adipose tissue. Perigonadal VAT was isolated and washed with ice-cold PBS. VAT was then minced to 2–3 mm pieces, added 4 mL of enzymatic digestion mix and transferred to gentleMACS C-tubes (130-096-334; Miltenyi Biotec, Bergisch Gladbach, Germany). Tissue was then dissociated using the gentleMACS Octo Dissociator (130-095-937; Miltenyi Biotec, Bergisch Gladbach, Germany), program name: mr_adipose_01, ran 3 times. Suspensions were subsequently filtered with a 100 μm cell strainer, washed with ice-cold PBS and stained for cell-sorting. Adipose enzymatic digestion mix contained 1 mg/mL bovine-serum albumin, 0.77 mg/mL Liberase (0541151001; Roche, Indianapolis, USA), 15.8 mU Hyaluronidase (H3506; Sigma-Aldrich, St. Louis, USA), 25 mU DNAse1 (DN25; Sigma-Aldrich, St. Louis, USA) and 1.5 μM Ca^2+^ in Hanks’ Balanced Salt solution.

### Immunohistochemistry

White adipose tissue was excised, fixed in formalin for 48 h, embedded in paraffin and 5 μm sections were generated. Sections were stained as previously described [24], using anti-F4/80 antibody (1:250, 70076; Cell Signaling Technology, Danvers, USA). The immunofluorescence analyses of multinucleated cells was performed using ImageJ (NIH, Bathesda, MD, USA).

### Flow Cytometry Sorting and Analysis

Single-cell suspensions were added a live/dead cell staining with blue reactive dye (1:250, L23105; Invitrogen (Thermo Fisher Scientific, Carlsbad, USA) and Brilliant Violet 510 anti-CD45 antibody (1:100, 103137; Biolegend, San Diego, USA) and isolated using the BD FACS Aria Iiu (BD Biosciences, San Jose, USA). During cell sorting, cellular debris were excluded with FSC and SSC gating and dead cells excluded with UVB channel negative selection. CD45^+^ cells were then positively selected and purified and processed for single-cell RNA sequencing as described in [25]. Flow-cytometric analysis was performed using the BD LSRII HTS (BD Biosciences, San Jose, USA). In addition to the live/dead and CD45 staining, Brilliant Violet 785 anti-Fcgr4 (1:100, 149535; Biolegend, San Diego, USA) and PE/Dazzle 594 anti-CD31 (1:100, 102429; Biolegend, San Diego, USA) antibodies were used. Data was analyzed using Flowjo version 10.4.2.

### Single-Cell RNA Sequencing

The sorted cells were then loaded onto a 10× Genomics Chromium instrument (10× Genomics, Pleasanton, USA), generating single-cell gel beads in emulsion (GEMs), 200,000 live CD45^+^ cells per experiment, and processed as described previously to sequence the 3’ end of transcripts [25].

### Read Alignment, Barcode de-Convolution, and UMI Counting

We used the Cell Ranger Single Cell Software Suite v. 2.2 to de-multiplex individual cells, process UMIs, and count UMIs per gene, following the standard pipeline and default parameters described at https://support.10xgenomics.com/single-cell-gene-expression/software/pipelines/latest/what-is-cell-ranger. Briefly, using *cellranger mkfastq* and *cellranger count*, FASTQ files were generated and aligned to the mm10 genome, sequencing reads were filtered by base-calling quality scores, and then cell barcodes and UMIs were assigned to each read in the FASTQ files. The mean read count was 79,505 reads per cell in the obese and 106,584 reads per cell in the CR sample. The filtered gene expression matrices were then used for downstream analyses for both obese and caloric restriction samples.

### Filtering of Cells

To identify low-quality cells and doublets, we looked at the distribution of the percent of mitochondrial genes expressed, the number of UMI in each cell, and the number of genes expressed in each cell. High outliers for the percent of mitochondrial genes expressed were removed, as high mitochondrial expression often indicates cells undergoing apoptosis. High outliers for the number of UMI per cell were also removed as possibly containing doublets, or multiple cells captured in a single GEM.

### Alignment of Datasets Using Canonical Correlation Analysis

After filtering the obese and caloric restriction scRNA-seq datasets that we generated, we merged these data with the Burl *et al.* [23] or Sharma *et al.* data, following the method outlined in Butler *et al.* [26]. Briefly, we identified the top 1000 variable genes in each dataset, used the intersection of the variable genes to perform canonical correlation analysis (CCA), and then aligned the canonical correlation vectors (CCs) using the R package Seurat [27]. In our analysis, we chose to align the first 25 CCs after examining the shared correlation strength as a function of the number of CCs for both sample (the Seurat function *MetageneBicorPlot*). We used the aligned CCs for downstream dimensionality reduction and clustering analyses.

### t-Stochastic Neighbor Embedding (t-SNE), Clustering Analysis, and Definition of Marker Genes

We used the first 25 aligned CCs to run t-SNE [28] dimensionality reduction and find Louvain clusters, using the default resolution of 0.6, using the R package Seurat [27]. For each cluster, we then defined marker genes, using cells from all 3 experiments to identify the top differentially expressed genes, requiring that marker genes for each cluster were expressed in at least 25% of cells in the cluster, and showed higher expression in that cluster than in the other cell populations.

### Data Filtering

Cells from all 3 conditions were merged using the RunMultiCCA function of Seurat [27], as described in Butler *et al.* [26]. The t-SNE procedure [28] was used to reduce the correlation vectors of all 3 samples to a 2-dimensional space, followed by unsupervised clustering. Three clusters present in obese and CR samples (comprising 2.5% and 18.6%, respectively, of all cells) were excluded from further analysis, since these were of non-hematopoietic origin and did not show expression of CD45 in the scRNA-seq data (data not shown). Although these cells likely arose from contamination during the cell-sorting procedure, they also may be non-hematopoietic cells that have surface CD45 expression, or possibly hematopoietic cells that transdifferentiated.

### Annotating Clusters Using Immgen

To assign clusters and individual cells to main cell types, we used the R package SingleR [29] and default parameters, using Immgen as the reference dataset and the parameter do.main.types = T. This resulted in the most likely main cell type being assigned to either each cluster, based on the average expression profile of the cluster, or each individual cell, using the expression profile of the cell.

### Differential Expression Analysis by Sample

We used Seurat [27] to identify differentially expressed genes by sample for each cluster, using the Wilcoxon test to generate *p*-values. To calculate log fold-change (logFC) values and *p*-values for all variable genes for each cluster, we used the following parameters: logfc.threshold = −Inf, min.pct = 0, min.cells.gene = 0, min.cells.group = 0, genes.use = my.seurat@var.genes. This allowed us to interrogate logFC values for clusters that did not have sufficient cell numbers to achieve statistical significance, but which showed significant differential expression in another cluster.

### Pseudotime Analysis and Branched Gene Expression Analysis

We used the R package Monocle [30] to reconstruct the divergence of cell lineages/trajectories in the cells identified as macrophages in our analysis. Briefly, we first used Monocle to estimate size factors, dispersion, and differential gene expression of the subset of macrophage cells, and then used the top 1000 most differentially expressed genes between the macrophage clusters to order cells in pseudotime. We then defined the branch with the largest proportion of monocytes as the root state of the tree, and plotted the trajectory of each sample (lean, obese, and CR) along the same pseudotime trajectory.

We used the BEAM feature of Monocle to define genes that show significant divergent expression across each branch point in the pseudotime analysis, using default parameters. We then used the top 100 significantly branching genes to interrogate divergent expression patterns across each branch point, and clustering the expression patterns into 4 main types for each branch point.

### GO and KEGG Enrichment Analysis

We used the R package ClusterProfiler [31] to look for enriched functions in the marker genes for each cluster, as well as differentially expressed genes by sample. Specifically, we used the enrichGO and enrichKEGG functions to look for terms that were enriched in particular clusters, and the compareClusters function to look for terms that showed differential enrichment across clusters.

Additionally, we used the online tool Gorilla [32] to cross-validate and further investigate enrichment of GO terms for specific clusters and differential expression analyses. We ran GOrilla using the “Target *vs* Background” option, using all genes with detectable expression in our dataset as the background dataset.

### Statistical Analyses

Data are expressed as mean ± SEM. When testing 3 groups, 1-way ANOVA with Tukey multiple comparisons testing was used, and 2-way ANOVA with Sidak multiple comparisons testing was used when comparing 2 parameters across multiple groups. *P* ≤ 0.05 was considered significant. Data was analyzed using GraphPad Prism 7.05.

## RESULTS AND DISCUSSION

### Single-Cell RNA Sequencing of Lean, Obese and Calorically Restricted VAT Reveals Distinct Subpopulations of Immune cells

Obese individuals have increased cardiovascular risk, and for the majority of them, this is the context of concurrent hyperlipidemia [33]. To identify immune cell (*i.e.*, leukocyte) populations in the VAT in different metabolic states, in a human relevant model, obesity and dyslipidemia were established for 24 weeks, after which VAT explants were obtained. To examine dynamic alterations of the adipose immune cell compartment induced by weight loss, an additional group of mice was calorically restricted following the HFD feeding, adapting a protocol originally reported in [13]. The daily food intake was measured on weeks 21–23 of the HFD feeding and the CR mice were then supplied daily with 70% of the same HFD for additional 2 weeks. Mice undergoing CR lost approximately 12% of their body weight (Supplementary Figure S1A), with ~25% decrease in VAT mass (Supplementary Figure S1B). As previously reported [13], CR promoted an increase in the crown-like structures in the VAT (Supplementary Figure S1C).

**Figure 1.**
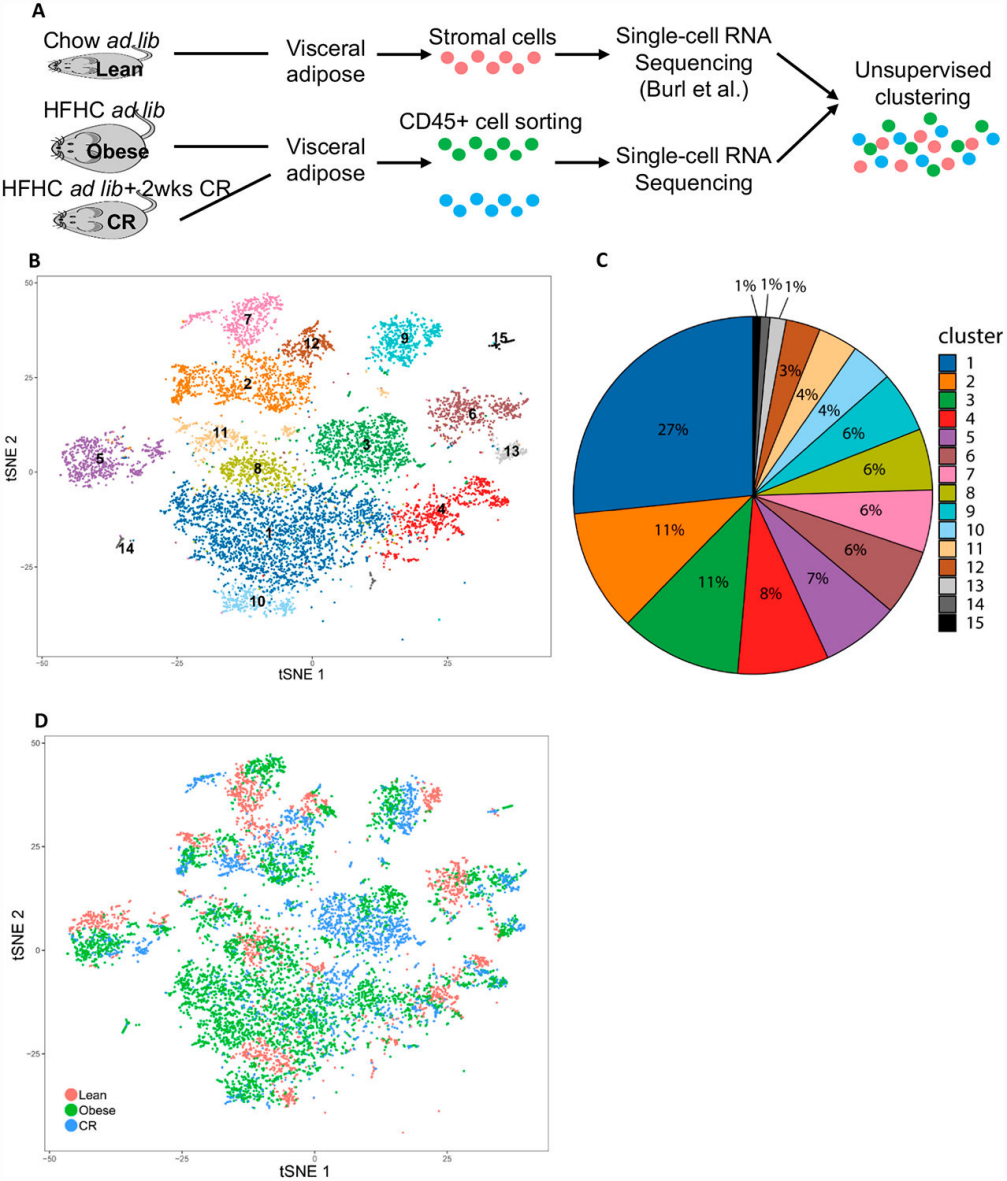
Single cell transcriptome analysis of mouse visceral adipose tissue leukocytes identifies 15 distinct subpopulations. (**A**) Diagram of experimental design. (**B**) t-Stochastic neighbor embedding (t-SNE) plot of 9958 VAT leukocytes from lean (reference [22]), obese and CR conditions, separated into 15 distinct clusters. (**C**) Overall proportion of leukocyte clusters in the VAT. (**D**) Representation of the t-SNE plot showing treatment of origin.

Viable CD45^+^ leukocytes were sorted from the VAT, and transcripts of individual cells were sequenced, using the 10× Genomics platform, following the method described in [25]. These data were merged with scRNA-seq data of lean VAT, published recently by Burl *et al.* [23] (Figure 1A). The numbers of cells that passed the quality control filtering were 2268 from lean, 5232 from obese, and 2458 from CR VAT (see Methods). Recent advances in scRNA-seq analysis allows for the parallel study of multiple datasets, obtained from separate models, treatments and even species [26].

After merging the 3 datasets (see Methods), we find that we can describe 15 distinct leukocyte populations (Figure 1B), of which the biggest cluster contained 27% of the total leukocytes in the VAT of the merged data (Figure 1C). Single cells from all 3 groups clustered together and showed a high degree of overlap (Figure 1D), indicating that our data merging strategy was successful. Nevertheless, one caveat of this study is that the lean group data were obtained using a different isolation protocol, using younger, normolipidemic mice. To investigate whether age or dyslipidemia significantly influences gene expression on a single-cell level, these data from lean and obese conditions were compared with age-matched lean and obese WT mice obtained from [34]. Results show substantial overlap in gene expression between the samples, according to the mice obesity state (Supplementary Figure S1D,E). Further study will be necessary to fully characterize the additional changes induced by hyperlipidemia, but the overall landscape of immune cells seems highly similar between the two conditions.

### Characterization of Leukocyte Heterogeneity in Cell Types and Their Putative Functions in the Visceral Adipose Tissue

**Figure 2.**
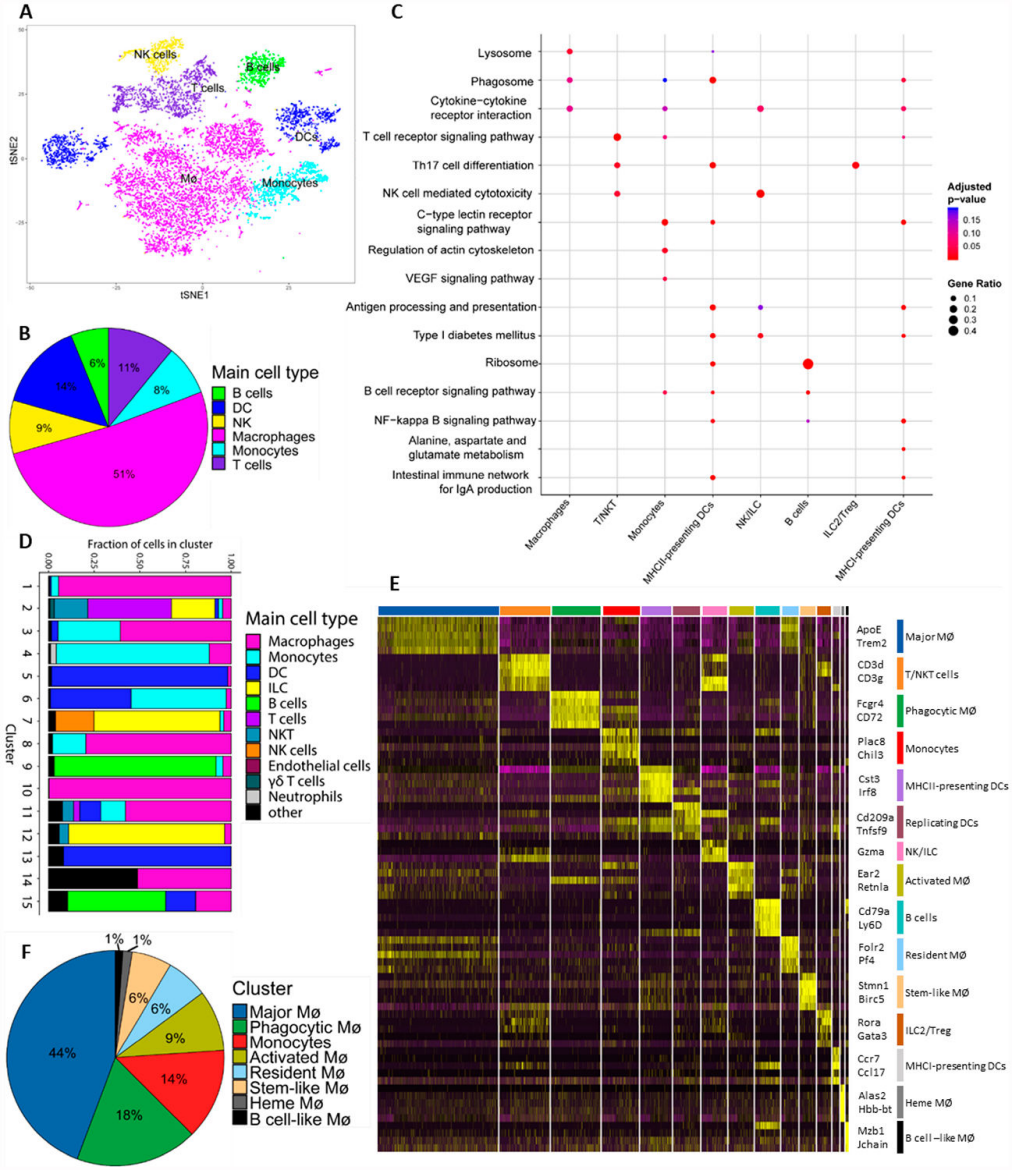
VAT leukocytes show functional and inter-cluster heterogeneity. (**A**) t-SNE representation and (**B**) proportion of the main VAT leukocyte cell types, assigned by SingleR, using average gene expression per cluster. (**C**) KEGG pathways of differentially expressed genes of different clusters. (**D**) Cell type distribution in each cluster, assigned by SingleR, using the expression profiles of individual cells. (**E**) Heatmap of the 5 most differentially expressed genes per cluster. (**F**) Proportion of monocytes/MØ in the VAT.

To characterize the main cell types of origin for the clusters, we used SingleR, which leverages the Immgen database to characterize cells by their closest match in an unsupervised manner [29] (Figure 2A). SingleR can run in multiple modes, the main distinction being whether the expression profile of each individual cell is compared to datasets available through Immgen, or whether the average expression profile of a cluster of cells is compared to Immgen. The comparison of average expression profile is equivalent to using expression data obtained by bulk RNA-seq and allows the identification of dominant, relatively large effects on a cluster. However, comparison of individual cell transcriptome can reveal intra-cluster heterogeneity. As a first pass at annotating our merged dataset, we used SingleR to identify the main cell type of each cluster by comparing the average expression profile of individual clusters. As expected, the majority of leukocytes in the VAT are MØs (51%, Figure 2B). Other leukocytes identified with this analysis are dendritic cells (DCs), T cells, NK cells, monocytes and B cells, comprising 14, 11, 9, 8 and 6% of the leukocytes in the VAT, respectively (Figure 2B). Of the 15 leukocyte subpopulations, SingleR assigned multiple clusters to several cell types, including 7 MØ clusters, 3 DC clusters, and 2 T cell clusters. NK cells, monocytes and B cells were each represented by a single cluster. These data suggest that mononuclear phagocytes are not only the most abundant, but also the most diverse in the VAT.

To examine the possible function of each unique subpopulation, we investigated gene ontology (GO) [35] and KEGG pathway [36] enrichment of the differentially expressed marker genes of each individual cluster (Figure 2C and Supplementary Figure S2A). Our data show that by and large, most leukocyte subpopulations have functions that are distinct from each other, but some pathways seem to be shared across multiple subpopulations. For instance, all mononuclear phagocytes had an enrichment in genes related to the phagosome, while the T cell clusters showed enrichment in Th17 differentiation (Figure 2C). In general, the data suggest that there is little functional overlap between the clusters, indicating that each subpopulation has a unique function.

To further interrogate the heterogeneity of cell types and functions within clusters, which may contain within them distinct subtypes of cells (as described in [29]), we again used SingleR, but this time annotated the main cell type of individual cells within each cluster, as opposed to the average expression of the entire cluster (Figure 2D). This analysis suggests that many clusters are not comprised of a single cell type, so that there is heterogeneity even within a given cluster. For instance, cluster 7, which was identified as NK cells when the expression of all cells was averaged, is comprised mainly (65%) of ILCs and only a minority (25%) of NK cells. This type of analysis may also indicate the origins of the different mononuclear phagocyte subpopulations. Of the 15 AT leukocyte subpopulations, 11 were identified as mononuclear phagocyte, of which clusters 5, 10, 13, 14 and 15 had no cells that were defined as monocytes, indicating they might not be monocyte-derived.

To further characterize the mononuclear phagocyte subpopulations, we examined gene expression of these clusters by identifying marker genes for each cluster, which can be defined as genes with the highest differential expression for a given cluster as compared to all other cells in the dataset. For instance, marker genes for MØs in cluster 10 include *Lyve1, Folr2, Klf2,* and *Gas6* (Figure 2E, Supplementary Table S1), which were all recently shown to be expressed in tissue-resident MØ from the heart [37], aorta [25] and AT [20]. This suggests that cluster 10 contains the resident MØ subpopulation, possibly explaining the absence of monocytes in that cluster. Accordingly, we postulate that clusters 3 and 6 are monocyte-derived, since a large proportion (>30%) of cells in those clusters are classified as monocytes by SingleR, whereas clusters 5, 10, 13, 14 and 15 are of non-monocytic origin. Interestingly, clusters 1, 8, and 11 have a small percent (<15%) of cells that are classified as monocytes. We postulate that cells in these clusters are of both monocyte-derived and of non-monocytic origin. Otherwise, it is plausible that these clusters are of monocytic origin, with cells in these subpopulations more rapidly differentiating or longer-lived in the adipose tissue. Another particularly interesting leukocyte subpopulation is cluster 15. When averaged gene expression was used to define the main cell type (Figure 2A,B), this subpopulation was assigned as MØs; however, when comparing the expression profiles of individual cells to the ImmGen database, it is apparent that most of the cells in cluster 15 are B cells. It was recently shown [38] that B cells can acquire MØ phenotype homeostatically and during inflammation, and it is possible that cluster 15 contains such MØ-like B cells. With that said, cluster 15 is the smallest of the clusters and therefore difficult to define marker genes with high statistical power, so in future studies we will analyze more cells to confirm the present findings.

Taking into consideration the information about cell type distribution in each cluster, as well as the differentially expressed genes and pathway analysis, we have assigned names to the different leukocyte subpopulations in our dataset (Figure 2E). Notably, we were unable to identify any eosinophil cluster, although this cell type was reported to be abundant (~5% of the stromal vascular fraction) in the lean VAT [39]. Hence, we searched for cells expressing the eosinophil marker SiglecF [39] (Supplementary Figure S2D) and found that cells expressing it are dispersed in different clusters and the abundance of the cells with detectable expression is generally low (a total of 0.64% of cells across all conditions). The absence of an eosinophil cluster might be due to a loss of AT eosinophils during obesity, a phenomenon that was previously described [20,39]. Another possibility is that in our conditions the eosinophil transcriptome was not unique enough to identify it as a separate cluster. It is also possible that there is a bias in the capture efficiency of different cell types with the scRNA-seq platform [40]. For example, some leukocyte subpopulations may be more sensitive to perturbations at certain steps of the VAT digestion/FACS/10× Genomics procedures than others, and do not survive. Nonetheless, our unique dataset allows the characterization of the leukocyte subpopulations that were captured in different metabolic conditions and subjected to identical procedures.

### MØ Heterogeneity in the VAT Show 7 Subpopulations with Distinct Inflammatory and Metabolic Functions

Single-cell analysis showed that ATMs are both the most abundant and the most heterogeneous population, with 7 of 15 clusters (Major, Phagocytic, Activated, Resident, Stem-like, Heme and MØ-like B cells) identified as MØs and another monocytic cluster (cluster number 4). We therefore decided to focus our next set of analyses on the ATM subpopulations.

VAT MØs were previously shown to participate in metabolic processes in (e.g., recycling the lipids secreted by adipocytes [41], producing Igf1 to regulate fat mass [42], and promoting thermogenesis [11]). Indeed, the KEGG and GO analysis of our data show that VAT MØs were enriched in many pathways related to metabolic processes, especially of lipids, such as oxidative phosphorylation, glycerolipid metabolism, and arachidonic acid metabolism (Supplementary Figure S2B, C). Similar to the non-MØ subpopulations in our dataset, the MØ clusters seemed to have mainly non-overlapping functions, demonstrated by uniquely enriched KEGG pathways (Supplementary Figure S2B) and GO terms (Supplementary Figure S2C).

Taken together, these data suggest that different MØ clusters have distinct metabolic functions in the VAT. Another interesting observation is that several clusters found in our VAT scRNA-seq dataset share transcriptional profiles with MØs that we recently described from atherosclerotic lesions [25]. Specifically, Trem2^hi^, Retnla^hi^Ear2^hi^, Folr2^hi^, IFN signature^hi^ and Ebf^hi^CD79a^hi^ populations previously described by our group share many genes with the Major MØ, Activated MØ, Resident MØ, Monocytes and B cell clusters, respectively (data not shown). These observations require further investigation to interrogate the similarities and differences in leukocyte subpopulations between atherosclerotic plaques and VAT.

### Identification of the Major MØ Cluster and Its Expansion in Obesity

Of the ATM clusters, the most predominant is the Major MØ, accounting for almost half of the ATMs in our merged single-cell dataset (Figure 2F). We speculate that this subpopulation is mostly tissue-resident, of embryonic origin, and partially monocyte derived, since this cluster contains 5% monocytes, as assigned by SingleR (Figure 2D). Furthermore, gene expression of this cluster follows similar patterns as the Resident MØ cluster (Supplementary Table S1), with increased expression of markers associated with resident cells, such as *Folr2* and *Gas6* [37]. However, the Resident MØ cluster expresses these genes more robustly, while the Major MØs show higher expression of genes that are associated with lipid metabolism, such as *Apoe* and *Sepp1*, as well as genes associated with MHCII-related antigen presentation, for instance *H2-Eb1*, *H2-Aa* and *CD74* (Supplementary Table S1).

**Figure 3.**
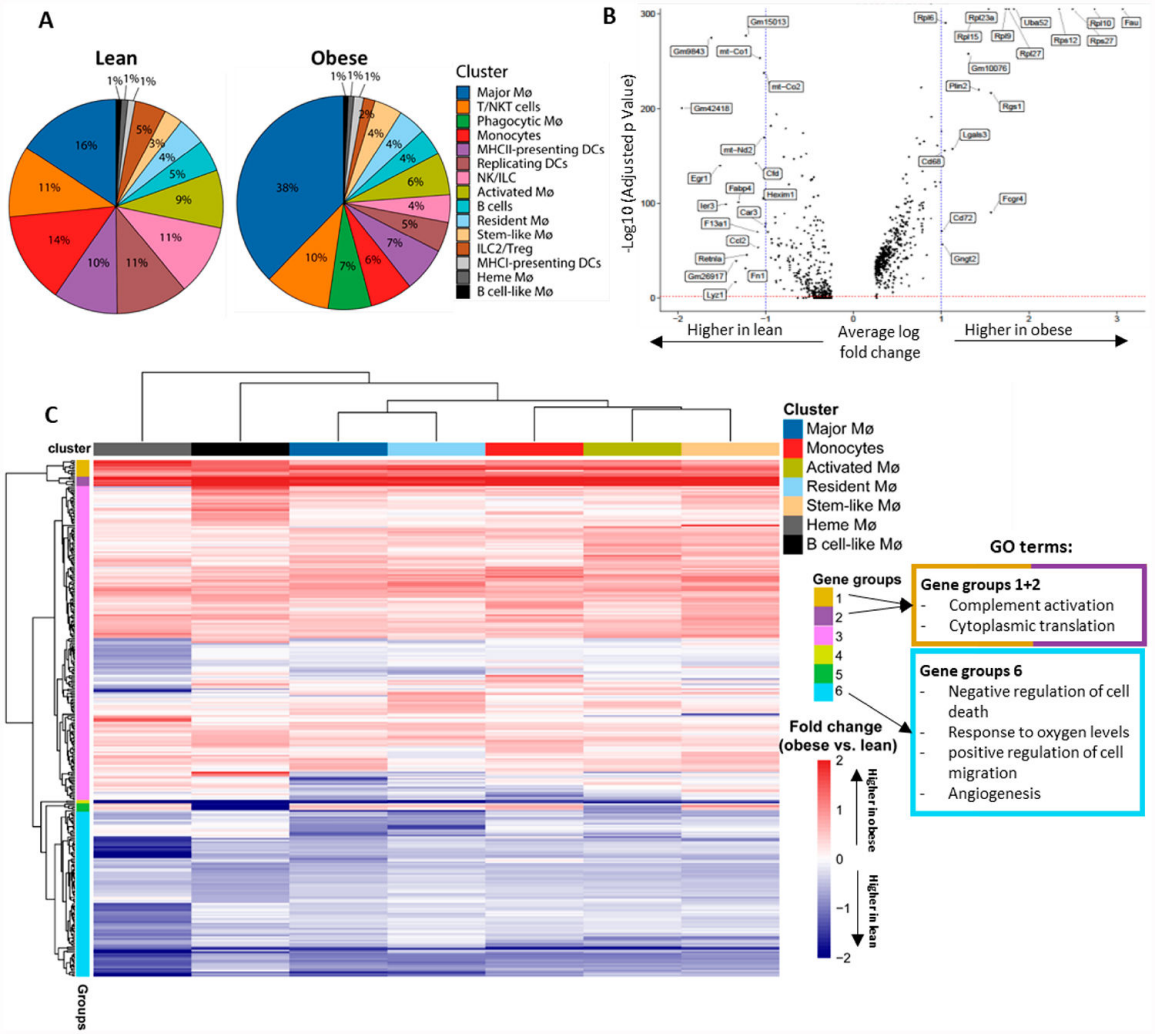
Obesity drastically alters both proportions and gene expression of VAT MØs. (**A**) Proportion of VAT leukocyte subtypes in lean (right) and obese (left) conditions. (**B**) Volcano plot of differentially expressed genes in MØs from obese versus lean VAT (*p*-adjusted < 0.05). (**C**) Heatmap of genes that are significantly differentially expressed per cluster between obese and lean conditions, including GO terms for gene groups that show the largest differential expression. The Phagocytic MØ subpopulation is absent in lean conditions and thus not included in this analysis.

It has been established in many previous studies that ATMs expand and dominate the obese VAT (reviewed in [1]); however, the specific characteristics of these MØs remain incomplete. To understand how obesity influences the VAT leukocyte population, and MØs specifically, we first examined the proportion of each cluster in lean versus obese conditions. Indeed, our data confirm the long-standing notion [1] that MØ abundance increases in the obese VAT (Figure 3A). Strikingly, the obese adipose tissue shows a dramatic increase in the Major MØ subpopulation (16% in lean *vs* 38% in obese, Figure 3A). Another noticeable difference is the acquisition of a MØ subpopulation in the obese state that is absent in lean VAT (Figure 3A). Investigation of KEGG pathways enriched in marker genes for this particular MØ subtype was not insightful; thus, in order to identify the possible function of this subpopulation, we used Gorilla [32], another program for GO terms identification. This program calculates GO term enrichments from a gene list, compared to a background list provided by the user, increasing the specificity of the search [32].

In our analysis, we compared the marker genes for this MØ subpopulation in obese VAT, but absent in lean VAT, to a baseline gene list that contained genes with detectable expression in our dataset that were not differentially expressed in any of the MØ subpopulations. Results of this analysis included GO terms involved in phagocytosis (“regulation of phagocytosis”, GO:0050764, *p*-value = 3.84 × 10^−10^) and endocytosis (“regulation of endocytosis”, GO:0030100, *p*-value = 4.53 × 10^−10^), hence we named this subpopulation Phagocytic MØs (Supplementary Table S2). Genes expressed by this MØ subpopulation involved in phagocytosis/endocytosis include *Fcgr4, Pecam1, Axl, Pycard, Fcer1g* (for more, please see Supplementary Table S3).

Interestingly, most other MØ subpopulations remained proportionally similar when comparing the lean and obese VAT, while the majority of the other leukocyte subtypes diminished. We speculate that there is an interaction between the different MØ subtypes that governs their abundances in VAT and that the expansion of the Major and Phagocytic MØs results in a proportional decrease in other leukocyte subtypes, such as ILC2/Treg, NK and DCs. Another possibility is that the absolute numbers of non-MØ subpopulations do not change, but the increase in MØ abundance results in their proportional decrease. From the single-cell RNA data alone, we cannot determine whether the absolute number of these leukocytes was different between the lean and obese state, since the total cell number captured in the 10× Genomics platform and passing quality control differs between experiments, and is assumed to be largely stochastic. Hence, all single-cell RNA sequencing experiments inherently result in estimates of proportion of cell type rather than estimates of absolute number of cells.

We also noted that obesity drives extensive transcriptional changes in VAT leukocytes (Figure 3B,C and Supplementary Figure S2A). To investigate whether obesity affects the same genes across multiple clusters, we investigated genes that showed at least a 1.5 fold increase or decrease in expression in any of the leukocyte subtypes in obesity relative to lean VAT. We then calculated log fold-change values for these genes in all of the leukocyte subtypes, and used hierarchical clustering to identify patterns of expression changes across cell types. Generally, leukocyte subpopulations showed significant overlap in the pathways transcriptionally modulated during obesity (Figure 3C and Supplementary Figure S2A). Heatmaps for the MØ (Figure 3C) and non-MØ (Supplementary Figure S2A) subpopulations show that genes that show the greatest change in expression in obese as compared to lean mice are shared across most or all clusters. However, genes with more moderate changes in obesity tend to differ in just a few clusters, but retain similar trends of expression across clusters. For example, in the MØ subpopulations, genes that are highly upregulated during obesity are associated with cytoplasmic protein translation (Figure 3C), which is similar to non-MØ clusters (Supplementary Figure S2A). Other genes upregulated during obesity in the non-MØ subpopulations are associated with response to interferon-ɣ (IFNγ) and antigen processing and presentation. IFNγ was previously implicated in worsening of obesity-related AT inflammation [43–45]. However, since our results show that many clusters upregulate IFNγ response genes it is important to investigate IFNγ effects on specific VAT leukocytes, to understand the contribution of this pathway per cell type. This analysis did not include the Phagocytic MØ subpopulation, which was absent in the lean VAT.

Genes that were downregulated in obese ATMs are associated with positive regulation of cell migration. This is consistent with previous studies [46,47], showing that obesity enhances the retention of MØs in the VAT. Finally, by performing scRNA-seq analysis in obese VAT leukocytes we hoped to distinguish between subpopulations that alter the metabolic phenotype versus those that influence the inflammatory phenotype. However, our data suggest that in a given single-cell subpopulation, genes related to both inflammation and metabolism are altered, indicating that many molecular pathways respond to obesity in each subpopulation of cells. Thus, we hypothesize that the dysregulation in the proportion of different leukocyte subpopulations in obesity could play a major role in both the inflammatory and metabolic responses.

### Short Term Caloric Restriction Following Obesity Induces Partial Recovery of Leukocyte Population Proportions to Those in the Lean State

After investigating the gene expression response to obesity on a single-cell level, we next interrogated the influence of CR-induced weight loss on VAT leukocyte subpopulations. As described previously [13], after the establishment of diet-induced obesity, a group of mice were calorie restricted by providing them daily (for a total of 2 weeks) with 70% of their *ad-libitum* HFD consumption. Importantly, the diet composition itself was unaltered, to ensure that any changes observed are due to the CR and not other factors, such as dietary fat content or other macro or micro-nutrients.

**Figure 4.**
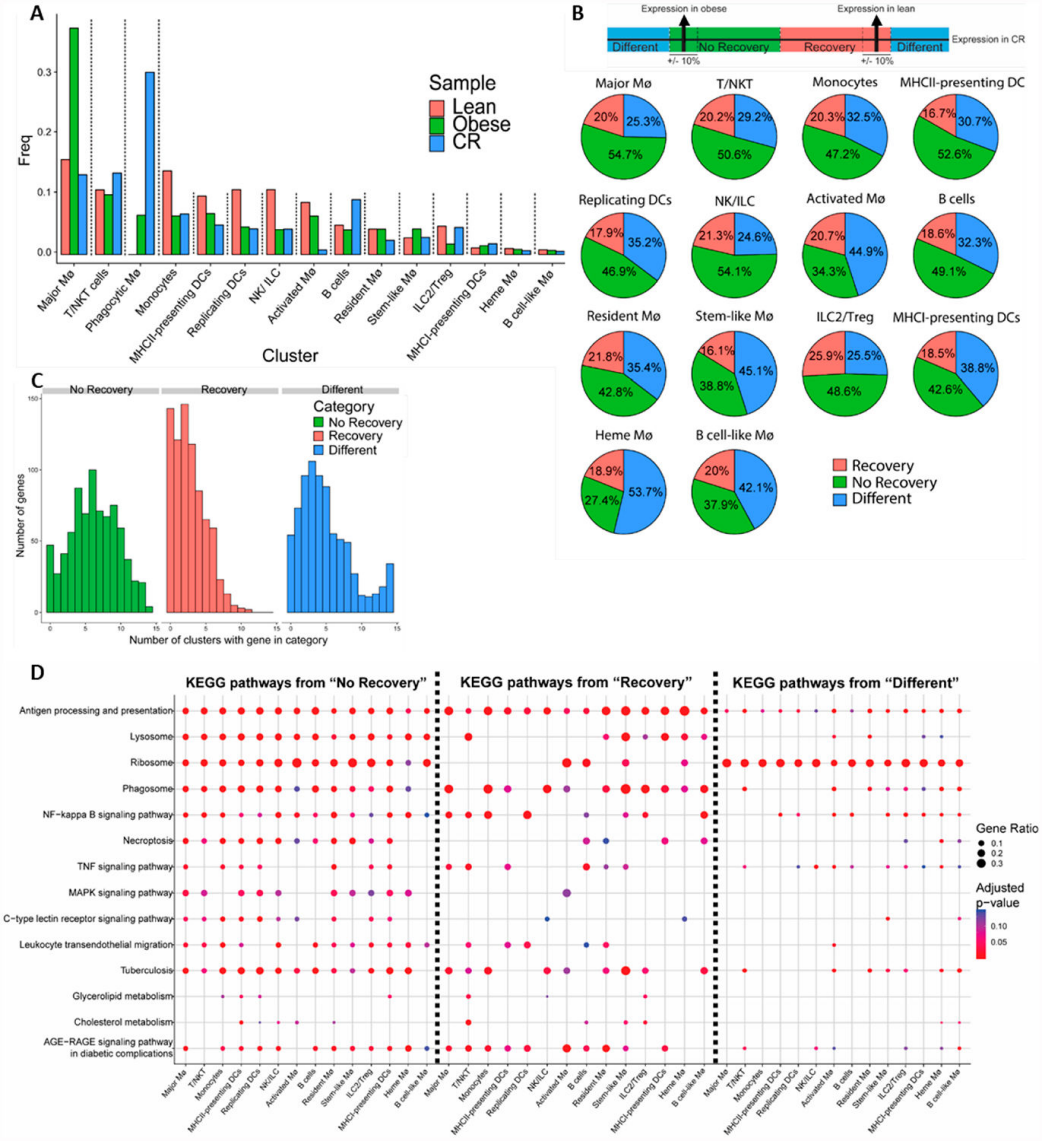
Short caloric restriction reverts some leukocyte subpopulations back to lean proportions, while gene expression remains similar to the obese state. (**A**) Distribution of VAT leukocyte subtypes in lean (red), obese (green), and CR (blue) conditions. (**B**) Stratification of genes whose expression was recovered (red) or not recovered (green) to the lean expression pattern following CR, or showed an expression pattern that is >10% different from either lean or obese (blue). The Phagocytic MØ subpopulation is absent in lean conditions and thus not included in this analysis. Schematic of the stratification strategy (top). (**C**) Distribution of the number of clusters that share the expression pattern of differentially expressed genes in each of the recovery gene groups. (**D**) KEGG pathways significantly enriched in each of the gene recovery states.

The most striking difference in the CR VAT is the accumulation of the Phagocytic MØ subtype. This subpopulation, which is absent in the lean condition, is the largest immune subpopulation in CR VAT, comprising 30% of CR leukocytes, versus only 7% in the obese VAT (Figure 4A). As in other clusters, Phagocytic MØs show transcriptional changes between the obese and CR treatments (Supplementary Figure S4A). It has been previously reported that upon CR there is an initial increase in ATMs, followed by a gradual decrease, until the VAT MØ content resembles lean proportions [13]. Our study now shows that in addition to alteration in abundances, there is a change in the “flavors” of CR ATMs. It will, thus, be interesting to investigate dynamic changes in the leukocyte subpopulations over longer periods of time post-CR, to determine whether there are remnants of obesity or CR. In humans, an increase in subcutaneous ATM content was reported following extreme hypocaloric diet [14]. It will be interesting, then, to interrogate whether the Phagocytic MØs described herein are similar to the ones that accumulate in human AT.

Notably, examination of the proportions of the different VAT leukocyte subtypes post-CR revealed that 3 subpopulations (Major MØs, Stem-like MØs and ILC2/Treg) reverted back to lean VAT frequencies (Figure 4A). Diminished VAT Treg was reported to associate with obesity and enhanced VAT inflammation [48], however, the effects of CR following obesity on Tregs have remained unclear in the literature. Our data suggest that CR restores VAT Tregs to lean proportions and we postulate that these cells are important for resolution of obesity-related inflammation. Interestingly, leukocyte subpopulations that decreased in obese VAT (Monocytes, MHCII-presenting DCs, Replicating DCs and NK/ILC) did not revert to lean proportions with CR. A third group of VAT leukocyte subtypes (Phagocytic MØs, Activated MØs, B cells and Resident MØs) showed unique proportions after CR that did not resemble lean or obese conditions.

### Gene Expression Following CR Primarily Remains Similar to the Obese State than the Lean State, with Genes That Are Recovered Involved in Antigen Presentation and Phagosome Pathways

Next, in addition to comparing leukocyte heterogeneity across the different groups, we questioned whether gene expression following CR was similar to obese VAT, or whether there was a recovery to lean conditions. To do so, we looked at the subset of 783 genes that showed statistically significant differential expression between at least one pairwise comparison of the samples (obese *vs* lean, CR *vs* lean, or CR *vs* obese) in at least one cluster.

We then characterized each gene in each cluster as either “recovered” from obesity (gene expression in CR is closer to lean than to obese), “not recovered” (gene expression in CR is closer to obese than lean), or “different” (gene expression in CR is more than 10% different from both obese and lean, and not intermediate to obese and lean, Figure 4B). This analysis does not include the Phagocytic MØ subpopulation, since it was absent in lean conditions. Our results show that across clusters, 16.1–25.9% of genes that were differentially expressed in obese VAT have recovered following CR and are expressed similarly in the CR condition to the lean condition (Figure 4B). Interestingly, the proportion of recovered genes is largely similar across all leukocyte subtypes. Accordingly, the largest proportion of genes in most clusters did not recover with CR and their expression is similar to that of the obese. This was to be expected, since CR was performed for only 2 weeks. Notably, a large proportion of genes in each cluster has a unique expression pattern in the CR condition that is not similar to either the obese or lean states (“Different”, Figure 4B).

We further investigated the genes that fall into the categories of “Recovery”, “No Recovery” and “Different”. To understand whether these genes change in a coordinated fashion, we first looked into the overlaps across clusters of genes in each of the categories (e.g., “Recovery”, “No Recovery” and “Different”). Our data show that most genes that did recover and reverted back to the lean state following CR did so in only one or a handful of clusters (Figure 4C). Conversely, for non-recovered genes (those that remained similar to the obese state), there was a nearly-normal distribution in the number of clusters in which a given gene remained similar to that of the obese state, showing that many of the non-recovered genes are shared among many clusters (Figure 4C). Finally, genes that show an expression level unique to the CR state (“Different”) showed a nearly bimodal pattern in cluster overlap, with most genes categorized as “Different” in just a few clusters, but an excess of genes that were categorized as “Different” in all or most clusters (most of which were ribosomal genes, Supplementary Figure S4B).

When investigating the pathways enriched in the “Recovery”, “No Recovery” and “Different” groups in each cluster, it was apparent that pathways were shared across many clusters (Figure 4D). Moreover, there were many overlapping pathways between the “Recovery”, “No Recovery” and “Different” gene sets (Figure 4D), with significant variation in the number of differentially expressed genes in each pathway (depicted in Figure 4D as the dot size). For instance, genes that recovered, did not recover, or showed divergent expression with CR, were all enriched for the “Antigen processing and presentation” and “Phagosome” pathways. However, the proportion of genes related to these pathways was largest in the “Recovery” and smallest in the “Different” group. The fact that the pathways are mostly shared between the “Recovery”, “No Recovery” and “Different” gene sets and across clusters suggest that these pathways are most sensitive to the metabolic alterations introduced by obesity and subsequent CR. Moreover, it has been previously reported that MØ phagocytosis is impaired in obesity [49]. Our data show that many phagosome related genes are recovering during CR, implying a restoration of this pathway toward normal capacity. Some other pathways in this analysis seemed to be more exclusive to the “No Recovery” group, such as the MAPK signaling and C-type lectin receptor signaling pathways, which may indicate that these pathways are less sensitive to CR.

After establishing that the pathways altered in CR are shared among clusters, we again examined whether individual gene expression changes are coordinated between clusters. Like the response to obesity, our data show that gene expression is altered similarly across multiple clusters (Supplementary Figure S4C). Genes that are downregulated in CR relative to obesity are associated with response to IFNγ and antigen processing and presentation. As noted above, these pathways were enriched in leukocytes in the obese state, and it seems that CR specifically reverts these changes, which goes together with processes of inflammation resolution. Additionally, relative to obesity, CR had enhanced expression of genes associated with lipid homeostasis and reverse cholesterol transport, which may point to an effort to clear excess lipids.

### Pseudotime Analysis Shows Distinct Trajectory for Major and Phagocytic MØs in Obesity and Following CR

**Figure 5.**
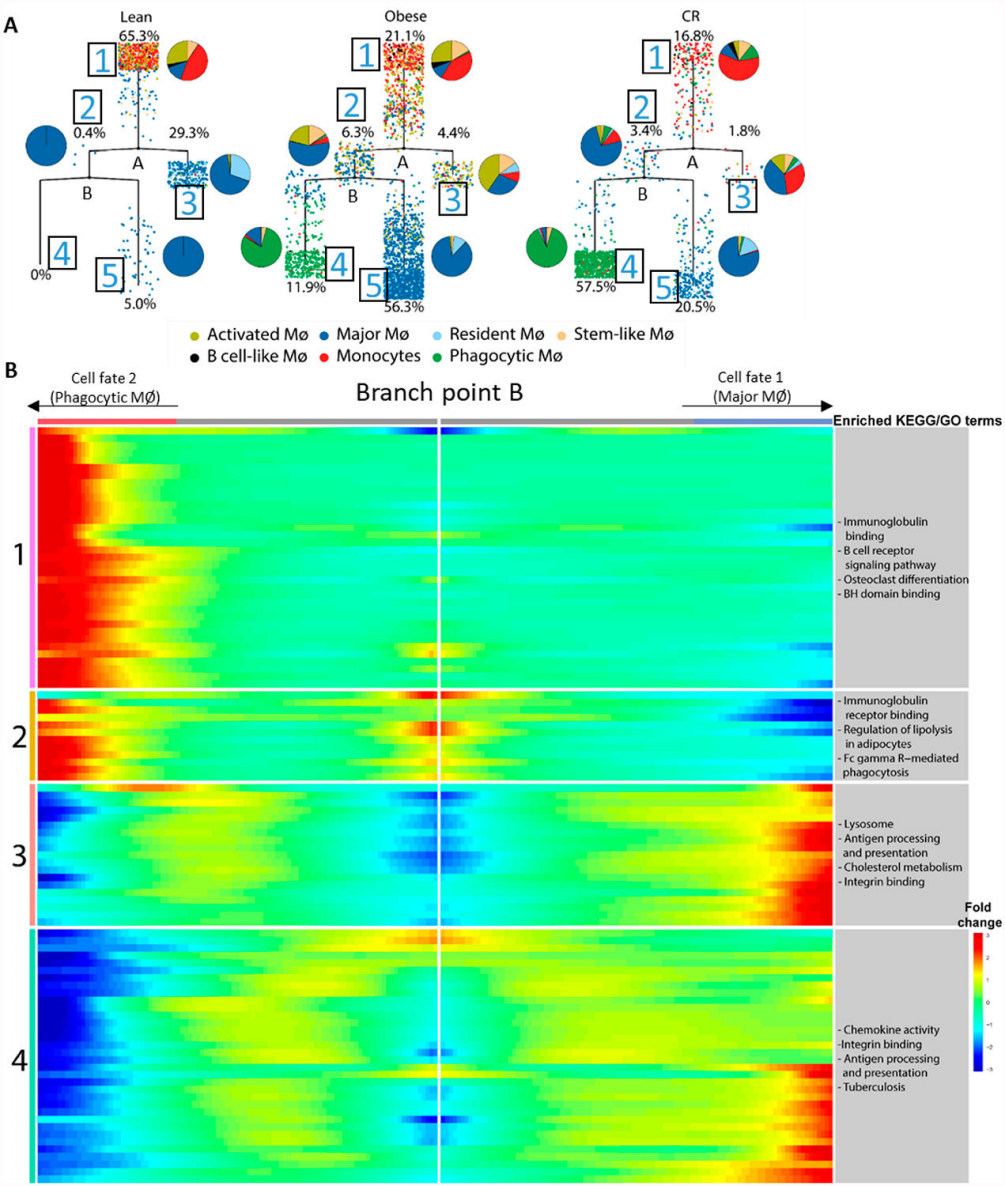
Pseudotime analysis shows distinct trajectories of the Major and Phagocytic MØs in obesity and caloric restriction. (**A**) Pseudotime analysis was performed using Monocle of the lean, obese and CR merged dataset for all monocytes/MØs. Monocytes were defined as the root population. Pie charts indicate the proportion of cells from each cluster that are assigned to each branch of the pseudotime trajectory. Percentages indicate the proportion of all monocytes/MØs that are assigned to each branch. (**B**) Top 100 genes that distinguish cells in branch point B of the pseudotime, with their related KEGG and GO terms.

So far, we have described that lean, obese and CR VAT are composed of similar leukocytic subtypes, with the exception of the Phagocytic MØs. The next question we wanted to address was whether obesity or CR influences VAT MØ fate or state. For that, pseudotime analysis was performed on our merged scRNA-seq dataset. This analysis treats scRNA-seq data as a snapshot of unsynchronized cells and arranges them on a virtual timeline, to understand their trajectory. Since many cells in our data were defined as monocytes by SingleR (Figure 2D), and these are the precursors for some of the VAT MØs, we defined the monocytes as the root population (depicted as 1 in Figure 5A).

Arranging the cells in psuedotemporal order revealed 2 major bifurcations in the lean state. The percentage of monocytes/MØ (from the total MØ per treatment), as well as the distribution of different MØ clusters in each branch are shown in Figure 5A. Our results show that in the lean state, but not the obese and CR, most of the cells (65.3%) were at the root (top), and many MØ subtypes constituted the root population, occupying similar pseudotime space, indicating comparable trajectory to monocytes. In the lean state there was only one other major trajectory, which consists of all the Resident MØs and most of the Major MØs. Genes associated with these 2 trajectories are shown in Supplementary Table S4.

As mentioned above, the Major MØs are possibly of mixed origin (embryonic and monocyte-derived) and we hypothesize that the seed population at the top consists of monocyte-derived cells, with the other trajectory being tissue-resident MØs. Interestingly, in the obese and CR states, the pseudotime trajectory was more complex, with 2 additional major branches (branches 4 and 5, Figure 5A). In contrast to lean VAT, in the obese and CR states most of the cells were further in pseudotime space from the root population. Notwithstanding, both obese and CR had similar trajectories, with varying cell distribution in the 2 branches missing in the lean VAT. The differences in cell distribution between obese and CR probably reflects the dominance of the Major and Phagocytic MØ subpopulations, respectively, in the two conditions. Furthermore, pseudotime analysis shows that the Major and Phagocytic MØ have distinct trajectories in both obese and CR conditions, and that the Resident MØs in obese and CR conditions show a pseudotemporal trajectory more similar to the Major MØs that is distinct from their trajectory in the lean condition.

To further examine the drivers of the distinct trajectories in obese versus CR MØ, we examined the genes and pathways that show branch-dependent expression across branch point B (Figure 5B, Supplementary Table S4). This analysis shows that among the pathways associated with the Metabolic MØ trajectory are lysosome, antigen-processing and presentation, and cholesterol metabolism, while the pathways associated with Phagocytic MØs are immunoglobulin binding and Regulation of lipolysis in adipocytes. It is, thus, possible that the Phagocytic MØ subpopulation is the one described by Kosteli *et al.* [13] as MØs that have enhanced lipolytic capacity in response to CR. Moreover, Kosteli *et al.* described a transient increase followed by a gradual decrease in VAT MØ content upon CR [13]. We speculate that these observed kinetic changes mainly reflect the recruitment of monocytes that become the Phagocytic MØs and disappearance of the Major MØs, and future studies will examine the dynamics of the different MØ subpopulations described here.

### Histological and Bioinformatic Validation of the Enrichment of Phagocytic MØs during CR

**Figure 6.**
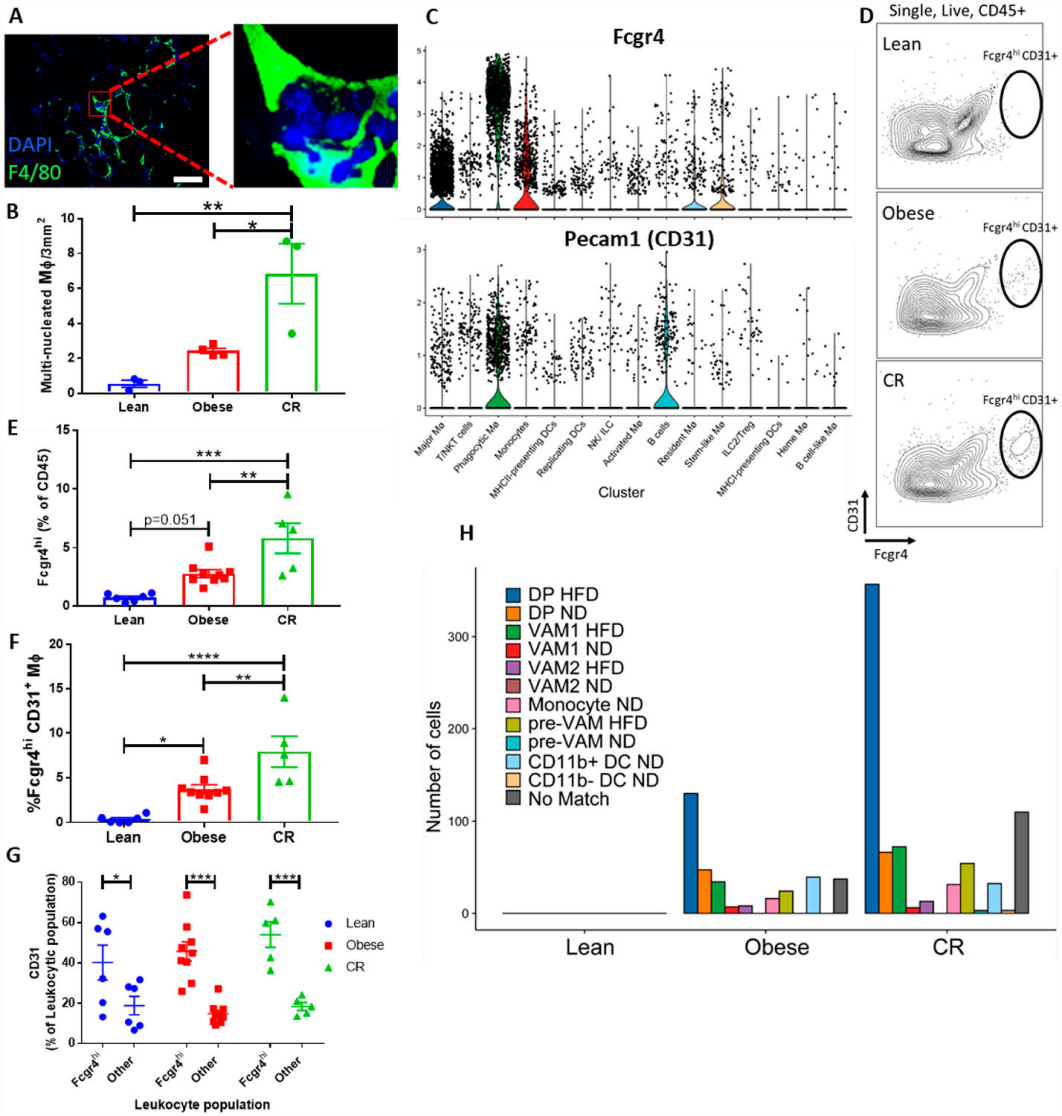
Validation of the presence of a specialized phagocytic MØ cluster. (**A**) Representative images of VAT stained with the MØ marker F4/80 (green) and a nuclear stain (DAPI, blue). Scale bar: 50 μm. (**B**) Quantification of multi-nucleated MØ (*n* = 3–4). (**C**) Violin plots showing increased expression of *Fcgr4* and *Pecam1* (CD31) in the Phagocytic MØ cluster. (**D**) Representative flow-cytometry counterplots of CD31 and Fcgr4^hi^ cells gated from single, live, CD45^+^ cells. (**E**,**F**) Quantification of the proportion of Fcgr^hi^ from (**E**) CD45^+^ or (**F**) CD11b + F4/80+ MØs in lean, obese and CR VAT (*n* = 6–9). (**G**) Proportion of CD31^+^ from either Fcgr^hi^ or all other leukocytes in the VAT. (**H**) Distribution of cells from the Phagocytic MØ cluster that significantly (*p* < 0.1) matched with populations from Silva HM *et al.* *p < 0.05, **p < 0.01, ***p < 0.001, ****p < 0.0001, using 1-way ANOVA with Tukey multiple comparisons testing (**B**,**E**,**F**) or 2-way ANOVA with Sidak multiple comparisons testing (**G**).

Although our data for the obese and CR VAT were merged successfully with the lean sample from Burl *et al.* [23], we were concerned about the absence of lean-derived cells in the Phagocytic MØ cluster. Furthermore, since the aforementioned cluster seems to be central in CR condition, we wanted to validate its existence. For that, we first sought evidence of enhanced phagocytosis by CR ATMs. Hence, VAT explants were sectioned, stained with the MØ marker F4/80 and quantified for the appearance of multi-nucleated cells. Multi-nucleated MØs may reflect that a MØ had engulfed other cell(s), however, MØ fusion was also shown to occur, to form multinucleated giant cells (MGC). The latter were shown to have increased abilities to phagocytose large particles [50]. In either case, the appearance of multinucleated cells would suggest an increase in phagocytosis. Images of VAT MØs clearly demonstrated the presence of multinucleated cells in CR and obesity (Figure 6A). Quantification of the multinucleated cells (Figure 6B) showed their accumulation in obese VAT and an even greater increase in CR. In contrast, lean adipose tissue had very few multinucleated cells.

To further validate the Phagocytic MØ subpopulation, we used the scRNA-seq dataset to define unique markers for this subpopulation. The most highly upregulated gene in this subpopulation was *Fcgr4*, which was also enriched in cells expressing *Pecam1* (CD31). Violin plots of these 2 genes (Figure 6C) indicate that they are candidate markers for the Phagocytic MØ subpopulation, with high expression of *Fcgr4* being a more specific marker of this cluster. Thus, we next examined the surface expression of Fcgr4 and CD31 in lean, obese and CR VAT leukocytes using flow-cytometry (Figure 6D,E). Our results show that lean VAT lacks the Fcgr4^hi^ population, but that it increases in obesity and it is largest in VAT from mice that were calorie-restricted following obesity (Figure 6D,E). Furthermore, the Fcgr4^hi^ population had enrichment of CD31 expressing cells, in comparison to other VAT leukocytes (Figure 6F).

Notably, the proportion of this subpopulation from all CD45^+^ cells identified using flow-cytometry is 2.8% in the obese group and 5.8% in CR group, which are also similar when gating from all MØs (Figure 6F). These proportions are markedly different from the ones seen in the scRNA-seq dataset, where this Phagocytic MØ subpopulation constitutes 7% of leukocytes in the obese condition and nearly 30% of leukocytes in the CR condition (Figures 3A and 4A). Flow-cytometry analysis of non-circular cells, which presumably also includes multinucleated cells, showed frequencies of Fcgr4^hi^ CD31^+^ MØs that resemble more those of the scRNA-seq than the proportions observed from the circular, single nucleus, events (1% in lean, 4.4% in obese and 16.8% in CR, Supplementary Figure S5A,B); however, still with substantial difference from the scRNA-seq data. This discrepancy may arise from the fact that not all the cells that have high *Fcgr4* transcripts also show high surface expression. Additionally, many of the cells in this cluster had high expression of *Fcgr4,* but not all, and the flow-cytometry analysis only captures the highly expressing cells. It is also possible that a bias in the capture efficiency of the scRNA-seq platform eliminates some populations from the single-cell analysis, thus enriching for populations that are captured. Partial validation of this point was obtained through flow-cytometry analysis of VAT MØ proportions in the lean, obese and CR conditions. Flow-cytometry data show somewhat decreased MØs, as compared with the proportions obtained from the scRNA-seq (Supplementary Figure S5C). Nonetheless, we find significant differences in the abundance of the Phagocytic MØ subpopulation between lean, obese and CR mice.

Finally, because of the potential biological significance of the Phagocytic MØ cluster, which may have a particularly important role in the remodeling of AT as it expands or rapidly contracts and the number of apoptotic cells that need to be cleared increase, we sought to confirm the identification of this cluster in an independent study. Recently, one of us described the heterogeneity of mononuclear phagocytes in mouse VAT, using surface markers to FACS-purify distinct populations and bulk RNA-seq of these populations was obtained (Silva *et al.* [20]). This paper mainly focused on resident MØs, which are tightly associated with the adipose vasculature; however, data were obtained for several subpopulations. Overall, RNA-seq data of 7 mononuclear phagocyte subpopulations defined by surface marker expression were acquired, some in both lean and obese states (after 20 weeks of HFD feeding). We, thus, used this bulk RNA-seq data and compared each cell from our dataset to the 7 purified populations, using SingleR [29]. Our results show that most cells from the Phagocytic MØ cluster were highly correlated with the HFD-derived double-positive (DPs) population from Silva *et al.* (Figure 6G). In their studies, Silva *et al.* found that the DPs are a monocyte-derived population (identified by high levels of CD11b, CD11c, CD64, and MHCII and intermediate levels of CD206), which was highly enriched post HFD feeding, corresponding well with our findings. In addition, replication of most leukocyte clusters identified in obese VAT was apparent in the dataset in [34], as shown in Supplementary Figure S5. In their study, Sharma *et al.* describe the effects of the loss of MØ netrin-1 on adipose tissue inflammation. They compared WT and myeloid-specific deletion of netrin-1 and showed with scRNA-seq that netrin-1 deficiency caused a 50% attrition of ATMs in HFD-fed mice (20 weeks), particularly of the resident MØ subset [34]. The overlap between the data described here and in Sharma *et al.* was substantial, with 91.1% of the lean and 90.1% of the obese cells significantly matching to our data.

## CONCLUDING REMARKS

It has been of great interest to determine the heterogeneity of the leukocyte populations in AT and their alterations at the molecular level in response to changes in their metabolic state, e.g., by HFD feeding or CR. In this report, we demonstrate the power of scRNA-seq to address these unresolved issues in AT biology. Our data show that there are 11 distinct mononuclear phagocyte clusters and an additional 4 lymphocyte clusters in VAT. Though further work will be needed to fully understand the specific contribution of each subpopulation to lean and obese VAT, it is clear that obesity promotes both inflammatory and metabolic alterations in a coordinated fashion in individual clusters and across leukocyte subtypes. Additionally, we found a novel specialized phagocytic MØ subpopulation, which is highly enriched following CR. We hypothesize that this subpopulation is responsible for clearing dead adipocytes and leukocytes, as well as lipid clearance, all contributing to limiting VAT inflammation and restoring a homeostatic state.

## Supplementary Material

Figure S1

Table S4b

Table S5

Figure S2

Figure S3

Figure S4

Figure S5

Table S1

Table S2

Table S3

Table S4a
