# Supplementary figures and images for: Single-Cell RNA Sequencing of Visceral Adipose Tissue Leukocytes Reveals that Caloric Restriction Following Obesity Promotes the Accumulation of a Distinct Macrophage Population with Features of Phagocytic Cells
Source: Immunometabolism. Author manuscript; Available in PMC 2019 Aug 8. (PMC6687332; doi:10.20900/immunometab20190008)

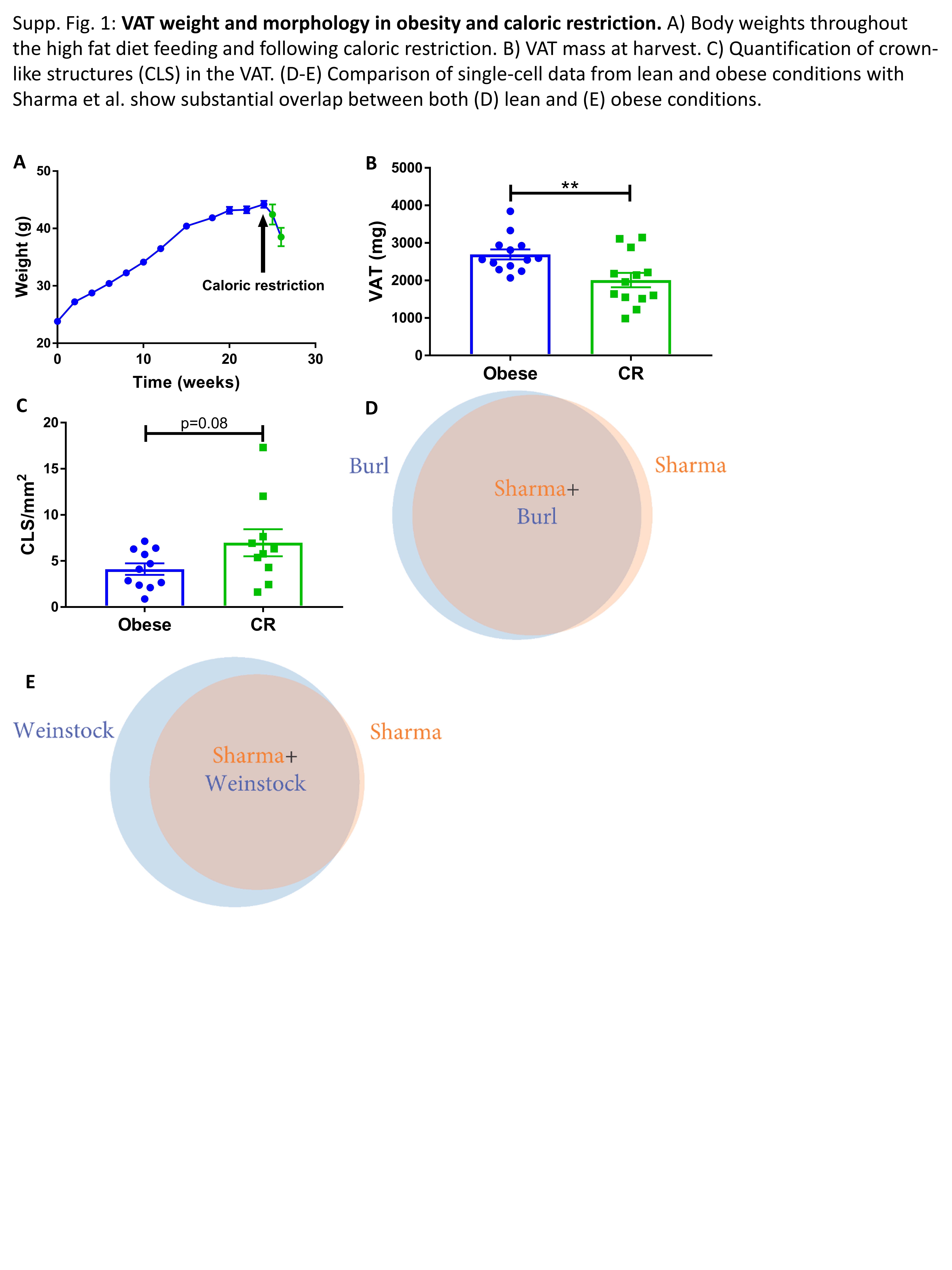

Supplement: Figure S1 [file NIHMS1042974-supplement-Figure_S1.JPG]

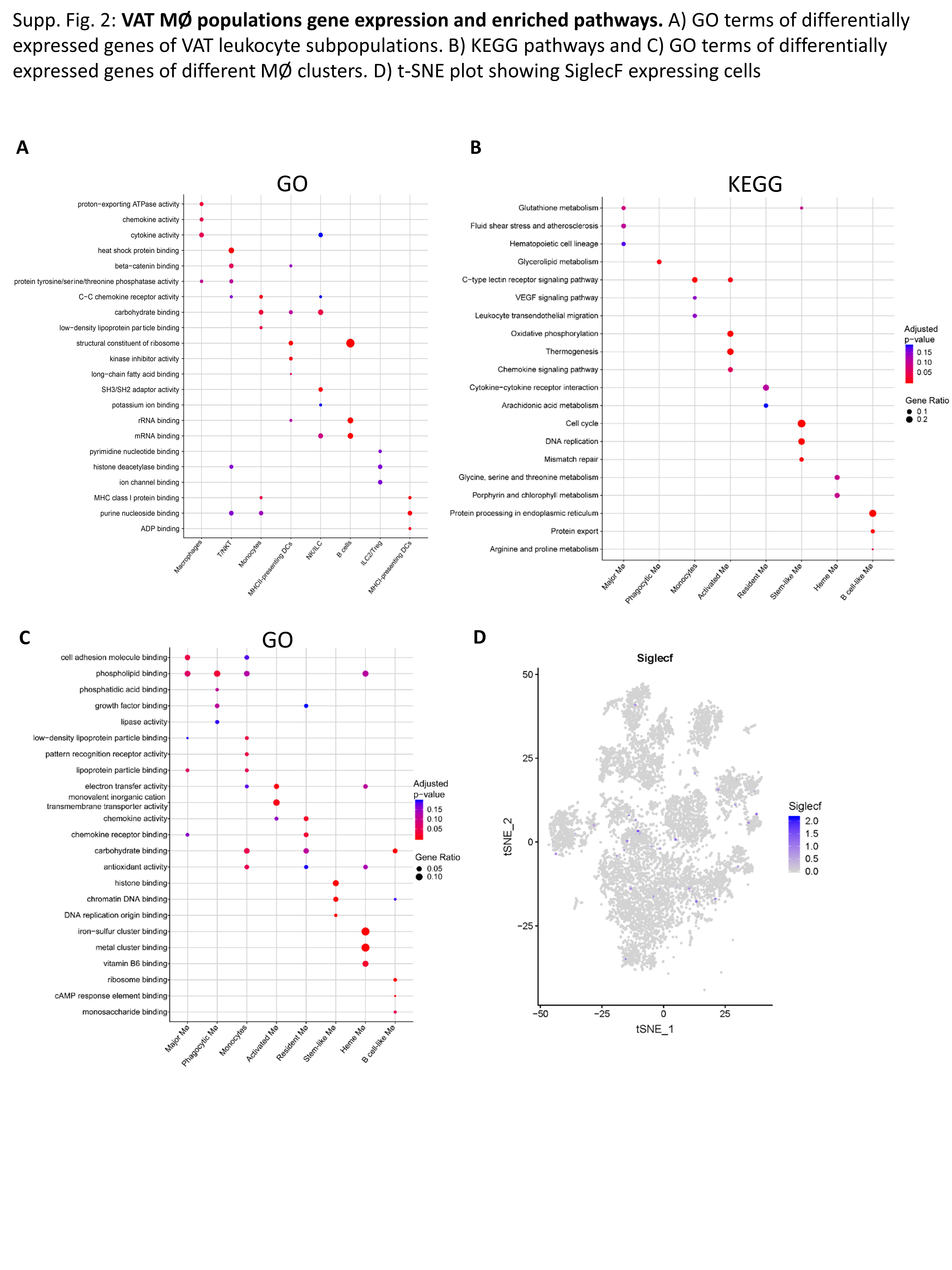

Supplement: Figure S2 [file NIHMS1042974-supplement-Figure_S2.JPG]

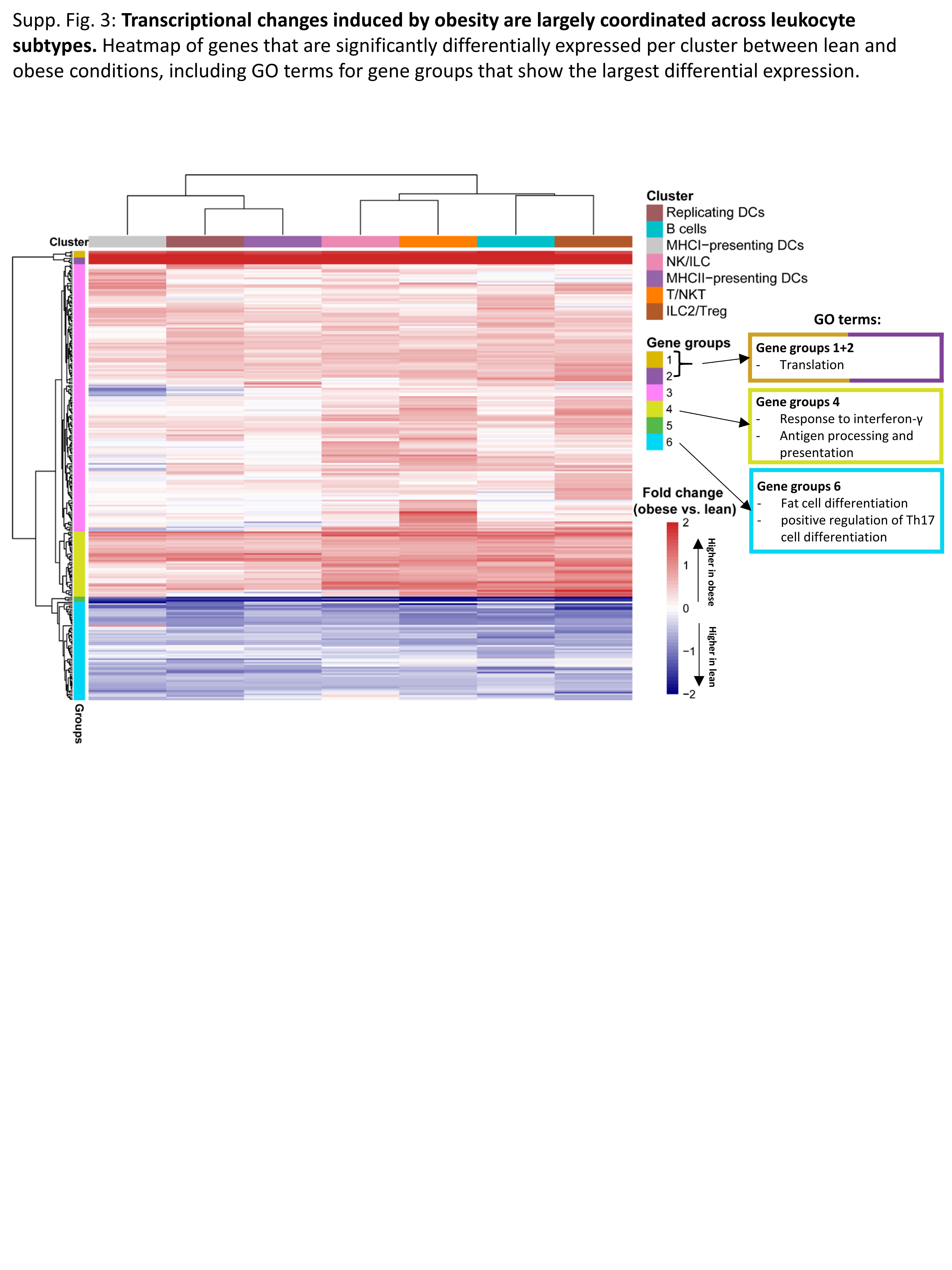

Supplement: Figure S3 [file NIHMS1042974-supplement-Figure_S3.JPG]

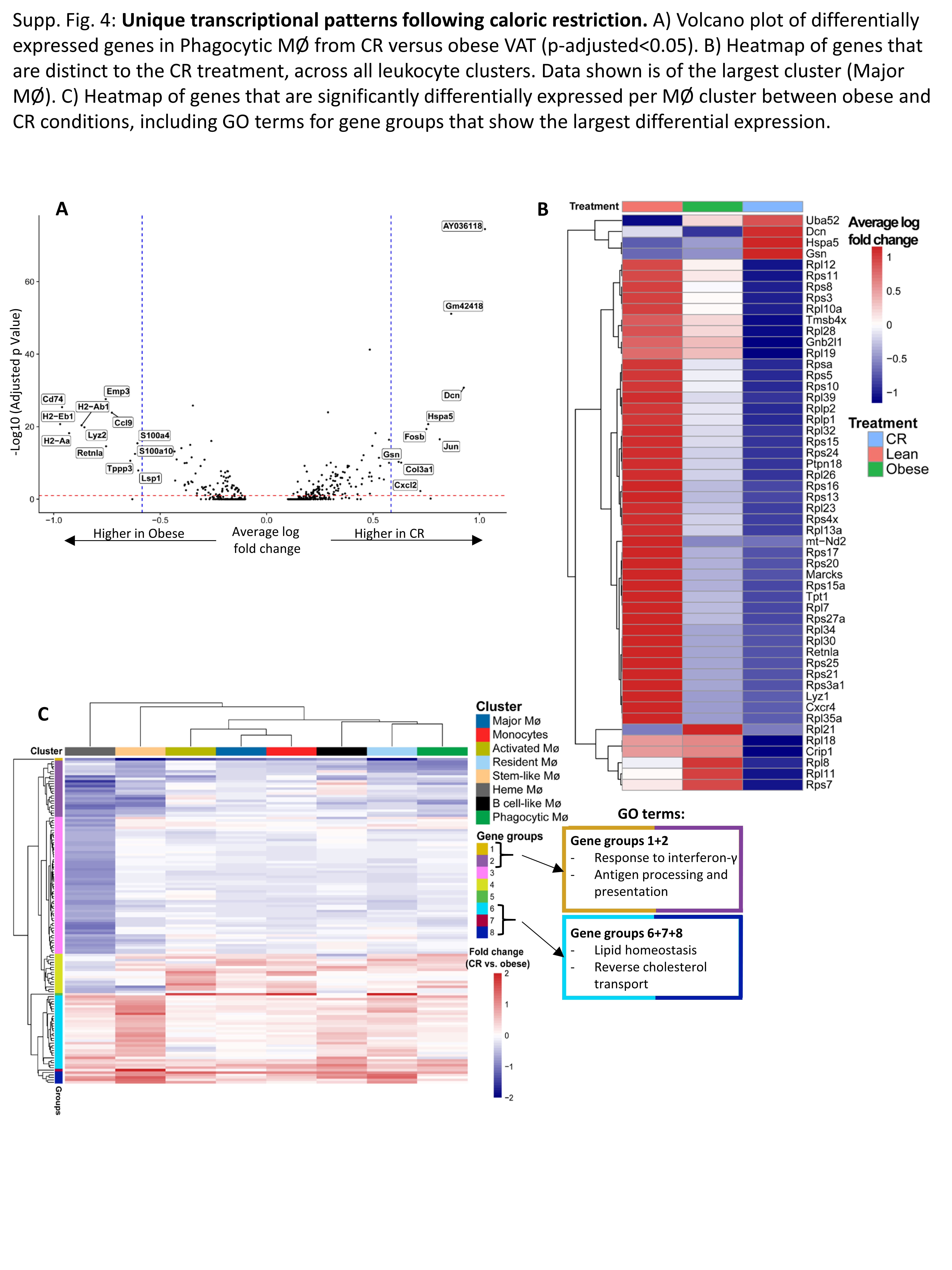

Supplement: Figure S4 [file NIHMS1042974-supplement-Figure_S4.jpg]

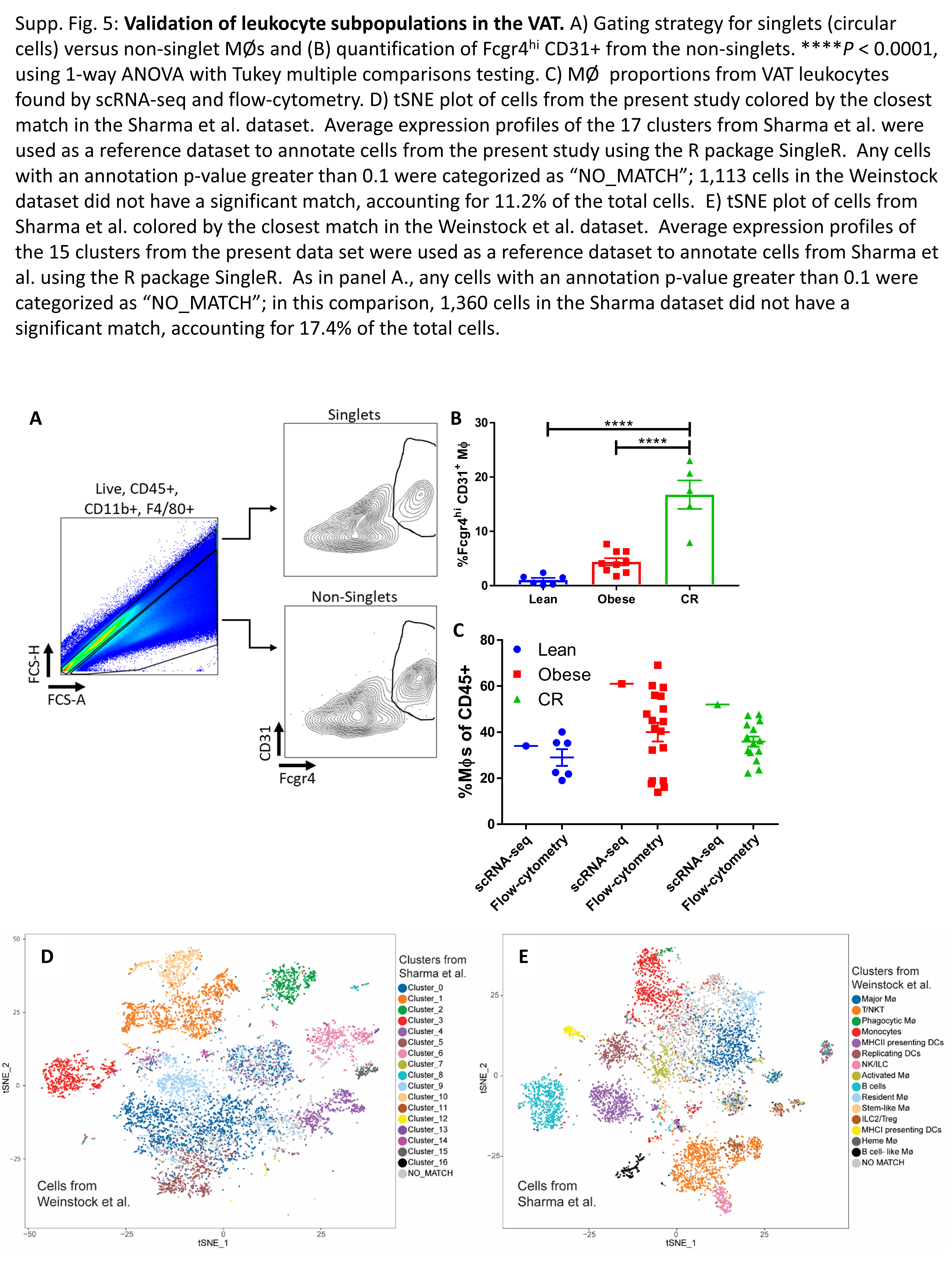

Supplement: Figure S5 [file NIHMS1042974-supplement-Figure_S5.JPG]
